# Rabies-based vaccine induces potent immune responses against Nipah virus

**DOI:** 10.1038/s41541-019-0109-5

**Published:** 2019-04-15

**Authors:** Rohan Keshwara, Thomas Shiels, Elena Postnikova, Drishya Kurup, Christoph Wirblich, Reed F. Johnson, Matthias J. Schnell

**Affiliations:** 1 Department of Microbiology and Immunology, Sidney Kimmel Medical College at Thomas Jefferson University, Philadelphia, PA 19107 USA; 2 Integrated Research Facility, National Institute of Allergy and Infectious Diseases, National Institutes of Health, Fort Detrick, MD 21702 USA; 3 Emerging Viral Pathogens Section, National Institute of Allergy and Infectious Diseases, National Institutes of Health, Bethesda, MD 20892 USA; 4 Jefferson Vaccine Center, Sidney Kimmel Medical College at Thomas Jefferson University, Philadelphia, PA 19107 USA

**Keywords:** Vaccines, Virology, Applied immunology, Infection, Infectious diseases

## Abstract

Nipah Virus (NiV) is a re-emerging zoonotic pathogen in the genus *Henipavirus* of the *Paramyxoviridae* family of viruses. NiV is endemic to Bangladesh and Malaysia and is highly fatal to both livestock and humans (human case fatality rate = 74.5%). Currently, there is no approved vaccine against NiV on the market. The goal of this study was to use a recombinant RABV vector expressing NiV glycoprotein (NiV G) to develop a bivalent candidate vaccine against NiV disease and rabies virus (RABV) disease, which is also a significant health burden in the regions where NiV is endemic. The rabies vector is a well-established vaccine strain that lacks neurovirulence and can stably expresses foreign antigens that are immunogenic in various animal models. Mice inoculated intranasally with the live recombinant RABV/NiV vaccine (NIPARAB) showed no signs of disease. To test the immunogenicity of the vaccine candidate, groups of C57BL/6 mice were immunized intramuscularly with a single dose of live vaccine particles or two doses of chemically inactivated viral particles. Both vaccination groups showed NiV G-specific seroconversion, and the inactivated (INAC) vaccine group yielded higher titers of NiV G-specific antibodies. Furthermore, cross-reactivity of NiV G-specific immune sera against Hendra virus (HeV), was confirmed by immunofluorescence (IF) and indirect ELISA against soluble recombinant HeV glycoprotein (HeV G). Both live and killed vaccines induced neutralizing antibodies. These results indicate that NIPARAB may be used as a killed virus vaccine to protect humans against NiV and RABV, and possibly as a preventative measure against HeV as well.

## Viral vaccines: bivalent vaccine immunizes mice against Nipah virus

The Nipah virus (NiV) infects livestock and humans with high mortality, but to date no vaccine has been approved that protects against NiV. Here, Matthias Schnell and colleagues developed a bivalent vaccine based on a highly immunogenic rabies virus vector and on expression of NiV glycoprotein. The vaccine led to the formation of neutralizing antibodies against the NiV glycoprotein in inoculated mice, which developed no disease as a consequence of immunization. Notably, the researchers also show that NiV neutralizing antibodies generated in the inoculated mice were cross-reactive to the Hendra virus, broadening the spectrum of the vaccine. The work provides a candidate for a NiV vaccine that uses a highly immunogenic vector and that has the potential to induce protection against not only NiV, but to rabies virus and Hendra virus as well.

## Introduction

Nipah disease is a highly fatal zoonotic disease whose causative agent is Nipah virus, a negative sense RNA virus of the *Henipavirus* genus within the *Paramyxoviridae* family. NiV was discovered in 1999 during an outbreak of encephalitis in Malaysia and was identified and isolated after methods for Japanese Encephalitis prevention were ineffective.^1^ This inaugural NiV outbreak caused at least 265 cases of encephalitis and 105 deaths, and necessitated the culling of over 1 million pigs, which were found to be important for transmission to humans.^1,2^ Although no further outbreaks in Malaysia have occurred, there have been 3 outbreaks in India and nearly annual outbreaks in Bangladesh since 2001, typically resulting from bat-to-human transmission via consumption of contaminated raw date palm sap.^3^ An outbreak in May 2018 in the state of Kerala in India had a case fatality rate of 86%, solidifying NiV as a persistent and grave threat in South Asia.^4^

Infections in fruit bats, the natural reservoir for the virus, seem to be asymptomatic. However, pigs can suffer from respiratory and neurological symptoms. Infection in humans is often highly fatal, and clinical manifestation is characterized by fever, headache, visual and motor skill dysfunction, acute respiratory illness, and encephalitis.^3^ The NiV strains that cause human cases in Bangladesh and India produce greater respiratory issues and more instances of human-to-human transmission than outbreak strains in Malaysia. As such, these cases are marked by higher mortality rates, reflective of pathogenic differences between the strains, as well as less developed healthcare infrastructure in the region.^3^

NiV attachment, fusion, and entry require coordinated effort from two membrane-anchored envelope proteins for successful infection. The glycoprotein (G) binds to ephrin-B2 and ephrin-B3 receptors on host target cells, and the fusion (F) protein is responsible for driving fusion of apposing viral and cellular membranes for entry.^5,6^ The ephrin-B2 and ephrin-B3 cellular receptors are highly conserved between potential host species (95–98% similar), including humans, horses, pigs, cats, dogs, and flying foxes, thus confirming the ability of NiV to infect a wide array of mammalian species.^5^ NiV G shows an affinity for ephrin-B2 and ephrin-B3 receptors that is 30-times higher than the glycoprotein of the closely related Hendra Virus (HeV), which has been known to cause outbreaks with severe respiratory disease in horses followed by transmission to humans.^7^ The strong neurotropism of NiV could be explained by the fact that ephrin-B2 and ephrin-B3 are highly expressed in the nervous system.^8^

NiV is classified as biosafety level 4 (BSL4) pathogen and considered to be a bioterrorism and agroterrorism threat.^9^ A safe and effective vaccine against NiV for both humans and livestock would be greatly beneficial to prevent NiV disease in endemic regions and to reduce the risk of NiV becoming a global danger.

Current work to establish a NiV vaccine has pursued various promising approaches with, some of which are outlined here. A live-attenuated recombinant measles virus (MV) expressing NiV G for human use (rMV-Ed-G) completely protected hamsters upon lethal NiV challenge. In a follow-up study, two African green monkeys (AGMs) immunized with 2 doses of rMV-Ed-G and subsequently challenged had no clinical signs before euthanasia.^10^ MV-based vaccine vectors can confer long-lasting immunity.^11^ Furthermore, preexisting immunity to MV in the human population does not seem to confound successful vaccination attempts.^12,13^ A VLP-based vaccine approach has also been tested for efficacy in a Syrian golden hamster challenge model. VLPs are antigenically similar to parental virus and are generally more immunogenic than subunit vaccines and can stimulate both humoral and cellular arms of the immune system.^14^ While both single and triple doses of VLPs expressing NiV G were able to fully protect hamsters from challenge, adjuvant was required to boost immunity and achieve high titers of neutralizing antibodies (nAbs). Moreover, incomplete lethality in the model allowed for survival in control groups not receiving vaccination, thereby complicating the vaccine-specific effect on survival.^15^ Further testing in pre-clinical models is warranted. A single administration of live-attenuated replication-competent recombinant vesicular stomatitis virus (VSV) lacking its native glycoprotein but expressing both Ebola GP and NiV G (rVSV-ZEBOV-GP-NiVG) was shown to prevent virus shedding, replication, and Nipah disease in AGMs.^16,17^ While live VSV-based vaccine vectors may have utility in emergency situations or for ring vaccination, regulatory approval for use in humans has been slow due to concerns regarding potential pathogenesis.^18,19^ Widespread adverse events and questionable efficacy in human populations of rVSV-EBOV may represent existing gaps in VSV-vectored vaccine approaches.^20–22^ However, replication-defective single-cycle recombinant vesicular stomatitis viruses (VSVΔG) pseudotyped with either NiV F or G showed protection against 1000 times LD_50_ NiV challenge in Syrian golden hamsters after a single dose inoculation, offering a distinct safety advantage over the live-attenuated rVSV-ZEBOV-GP-NiVG.^18^ Nonetheless, production requires multiple plasmid transfections which can be costly for large-scale manufacturing.

In this study, we aimed to develop a RABV-based vaccine against NiV for both animals and humans. Vaccines based on RABV have been shown to induce strong humoral responses against other pathogens and inactivated RABV vaccines are considered remarkably safe.^23–27^

## Results

### Rescue of NIPARAB in cell culture

The parental RABV vector used in this study, BNSP333, is derived from the SAD B19 strain, which is a vaccine strain attenuated by tissue culture passage.^26^ This vector was designed to contain an additional RABV stop-start transcription signal sequence with unique BsiWI and NheI restriction sites between the nucleoprotein (N) and phosphoprotein (P) genes for the introduction of foreign genes.^28^ To further increase the safety profile of the vector, an arginine to glutamic acid mutation was introduced at amino acid position 333 of RABV G, which greatly reduces neurovirulence.^23,29,30^

**Fig. 1:**
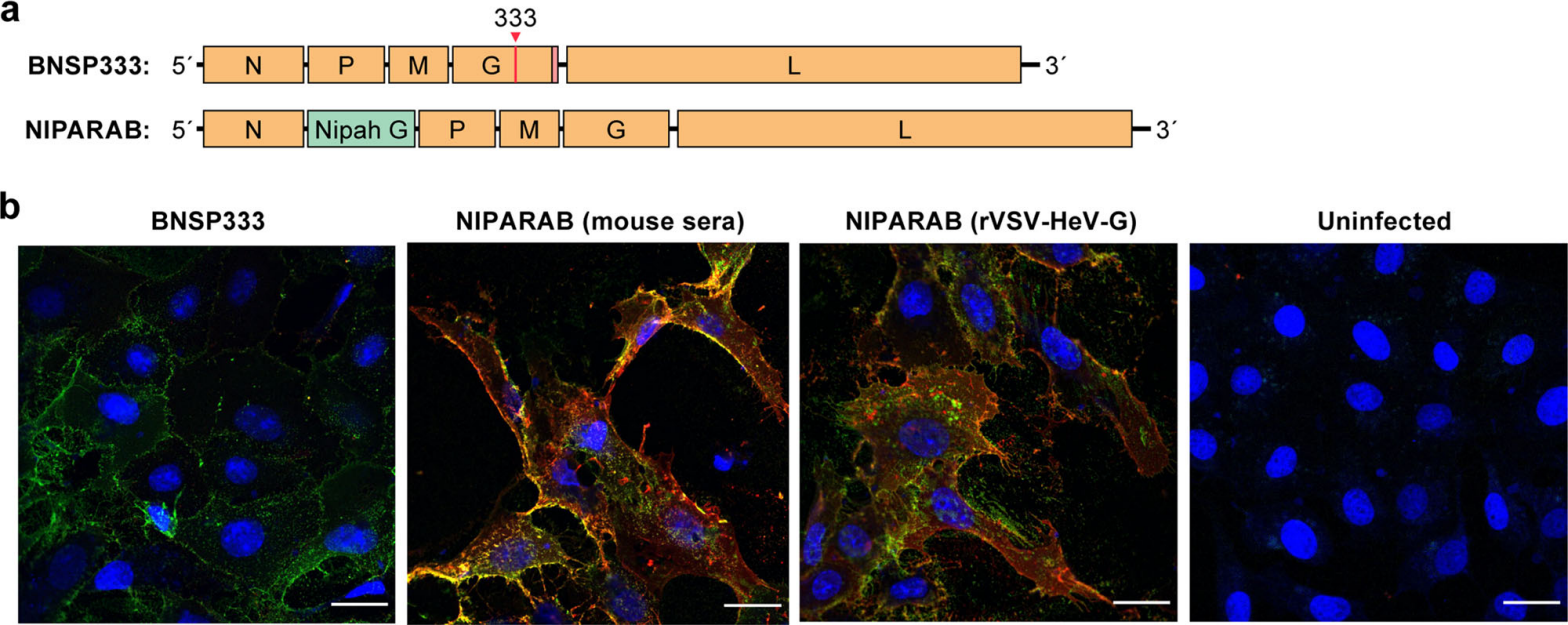
Vaccine constructs. **a** Schematic representation of recombinant rabies virus (attenuated SAD B19 vaccine strain) genome expressing codon-optimized Nipah virus glycoprotein gene (Bangladesh strain) in between the nucleoprotein (N) and phosphoprotein (P) genes of rabies. An asterisk indicates the Arg→Glu mutation in amino acid position 333 of RABV G that attenuates neurovirulence. Parental vector with no foreign gene insertion, used as a negative control in immunogenicity and pathogenicity studies, is also depicted. **b** Confocal microscopy image of VERO E6 cells that were ether mock infected or infected at an MOI of 0.01 for 48 h with live NIPARAB or BNSP333 before fixing and dual staining with a human monoclonal antibody directed against RABV G (green) and either polyclonal sera from mice immunized with INAC NIPARAB (pooled sera 45 days post-infection (dpi) from mice receiving 2 doses of 10ug each) or live rVSV-HeV-G (pooled sera 57 dpi from mice receiving one i.n. dose of 1E5 pfu) (red). Scale bars represent 24 µm

To develop full-length NIPARAB cDNA for use in recovery of the recombinant virus, we inserted a codon-optimized version of NiV G (Bangladesh strain) into the BNSP333 vector in between RABV N and P genes. This cDNA serves as the antigenome template from which single-stranded negative-sense RNA genomes are made. During recovery, this construct was co-transfected into BSR cells along with support plasmids individually bearing RABV genes required for assembly, packaging, and budding of recombinant virions, as previously described.^31^ For our studies, we generated both a live version of the vaccine and a chemically inactivated version using beta propiolactone which impedes virus replication while maintaining antigenicity of target immunogens^32^ (Fig. 1a).

### Expression of NiV G by NIPARAB

Successful antigenicity of NiV G and RABV G by our vaccine depends on their expression at the surface of NIPARAB-infected producer cells so that the host cell-derived envelopes of viral progeny will contain both glycoproteins. To analyze co-expression, VERO cells were either mock infected or infected with live BNSP333 or NIPARAB at a multiplicity of infection (MOI) of 0.01 for 48 h before performing a surface immunofluorescence assay followed by confocal microscopy imaging. Cells were dual stained with a human monoclonal antibody directed against RABV G and either pooled polyclonal necropsy sera from mice immunized with 2 doses of INAC NIPARAB or one intranasal (i.n.) dose of live recombinant VSV expressing Hendra G (rVSV-HeV-G). Hendra G-specific sera was used with the knowledge of cross-reactivity to identify NiV G expression in the absence of NiV G-specific sera. Only NIPARAB-infected cells co-expressed RABV G and NiV G (Fig. 1b), indicating that NIPARAB is a viable bivalent vaccine vector.

### Characterization of recombinant NIPARAB virions

**Fig. 2:**
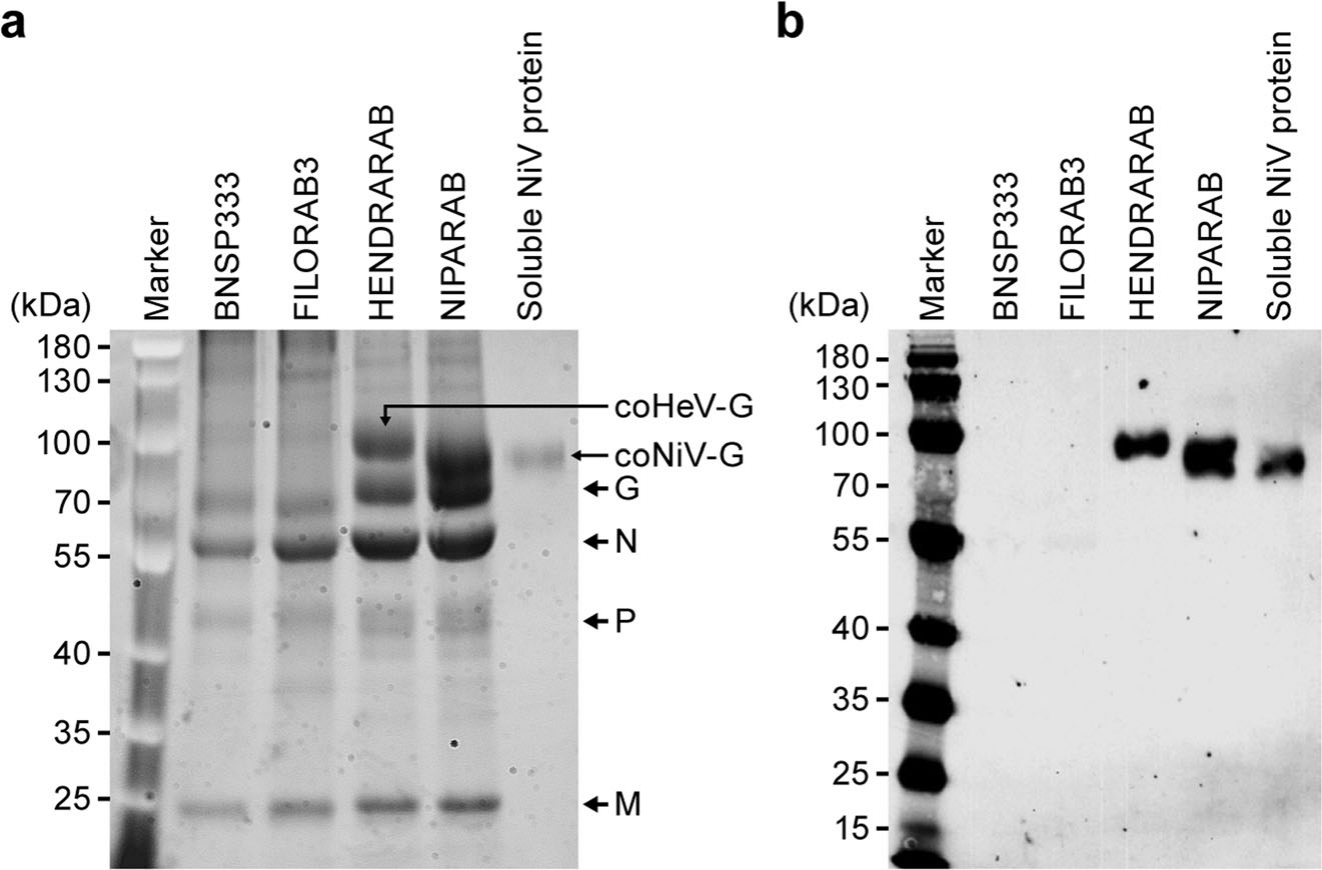
Analysis of purified virions of vaccine vectors. **a** Purified inactivated virions were loaded onto a 10% SDS-PAGE gel (4 μg per construct) and stained with SYPRO Ruby to visualize incorporated proteins. Critical RABV proteins as well as foreign glycoproteins are indicated. Soluble codon-optimized NiV G (0.5 μg) with transmembrane and cytoplasmic domain deletion (used for antibody capture in ELISAs) was loaded onto right-most lane for visualization. **b** Confirmation of NiV G incorporation into purified NIPARAB virions by Western blot analysis. 4 μg of purified inactivated NIPARAB or control virions were loaded onto 10%SDS-PAGE gel and transferred to a nitrocellulose membrane. The blot was probed with polyclonal rabbit anti-HeV G sera to identify NiV by cross-reactivity. Soluble codon-optimized NiV G (0.25 μg) with transmembrane and cytoplasmic domain deletion (used for antibody capture in ELISAs) was loaded onto right-most lane for visualization

To confirm that RABV G and NiV G are both incorporated into NIPARAB virions, we evaluated purified virions from several different recombinant viruses by 10% SDS-PAGE and SYPRO Ruby staining (Fig. 2). A prominent band with an apparent molecular size slightly above 70 kDa was exclusively detected in lanes containing INAC NIPARAB virions or soluble NiV G protein (Fig. 2). These bands were consistent with the expected size of NiV G,^33–35^ and they migrated similarly but distinctly lower than HeV G (~100 kDa) derived from virions of recombinant BNSP333 expressing codon-optimized HeV G (HENDRARAB).^7,36^ NIPARAB virions also incorporated all necessary RABV proteins as determined by comparison to parental BNSP333 virions.

Purified virions were also assessed by Western blot and probed with rabbit polyclonal HeV G antisera (Fig. 2b). Bands corresponding to HeV G (~100 kDa) and NiV G (>70 kDa) were specifically detected in lanes containing HENDRARAB and NIPARAB viral particles, respectively. Furthermore, soluble recombinant NiV G protein with transmembrane domain and N-terminal cytoplasmic tail deletion was also detected with a slightly lower molecular size than the full-length protein, as expected. This finding was consistent with results from the stained protein gel and signified that specific sera against HeV G could cross-react with NiV G. No proteins were detected with negative control virions from parental BNSP333 or recombinant BNSP333 expressing an unrelated glycoprotein from Marburg virus (FILORAB3). These results indicated that NiV G had successfully incorporated into the RABV vaccine vector.

### NiV G incorporation does not increase RABV vaccine pathogenicity

**Fig. 3:**
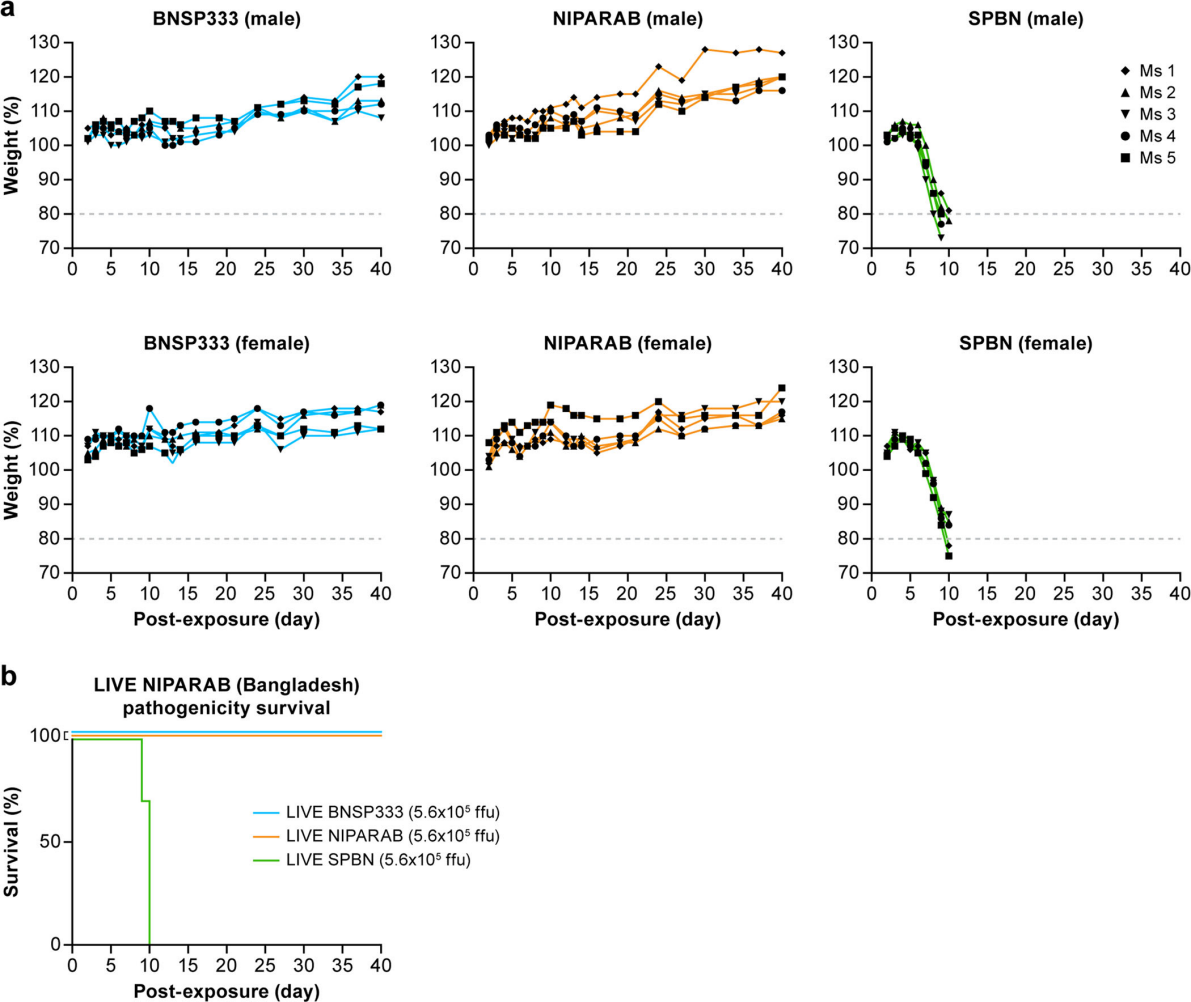
Pathogenicity of live NIPARAB vaccine in male and female B6 mice. **a** Groups of ten 6-week-old to 8-week-old C57BL/6 mice (5 males and 5 females per group) were intranasally (i.n.) infected with 5.6E5 ffu of live recombinant viruses: NIPARAB, BNSP333 (negative control), or SPBN (positive control). Mice were weighed daily and monitored for signs of disease until day 40 post-inoculation. Endpoint criteria for euthanasia were reached when mice lost more than 20% of their original body weight or displayed symptoms of disease. Weight curves are reported as percentage lost or gained over time from the baseline weight. **b** Survival curve of C57BL/6 mice in this study. The log-rank (Mantel-Cox) test was used for comparison of survival curves to assess significant differences in survival

While incorporating NiV G into the RABV vector does not trigger safety concerns in a killed vaccine, it could be an issue for use as a live viral vector. NiV is neurotropic, since its envelope protein binds to receptors predominantly expressed on neuronal cells, and Nipah disease has been known to cause encephalitis and neurological symptoms in infected humans.^1,3^ Therefore, live NIPARAB inoculation could potentially lead to pathogenicity by restoring neurovirulence, even though BNSP333 is nonvirulent after peripheral administration in adult mice.^23^ To study this potential impact, three groups of ten C56BL/6 mice (split equally by gender) were infected i.n. with 5.75 log_10_ focus-forming units (ffu) of either live BNSP333 (negative control), NIPARAB, or SPBN (positive control). Animals were monitored for weight loss and clinical signs of disease for 40 days to observe both immediate and latent adverse effects. Endpoint criteria for euthanasia were weight loss exceeding 20% of the original body weight or hallmark neurological symptoms, such as ataxia. SPBN is derived from the same rabies virus vaccine strain as BNSP333, but it lacks the attenuating mutation in RABV G, making it lethal when administered i.n. to adult mice.^23^ All mice receiving SPBN succumbed to infection by day 10 post-exposure, whereas all mice inoculated with BNSP333 remained healthy with no weight loss or malaise. Similarly, all mice infected with live NIPARAB survived the duration of the study with no apparent pathogenicity, as indicated by steady weight gain and the absence of neurological disease (Fig. 3a, b). These data demonstrate that addition of NiV G to BNSP333 does not reestablish pathogenicity in the parental vector within an immune competent mouse model and may be safe for use as a wildlife vaccine.

### Immunogenicity against NiV G

**Fig. 4:**
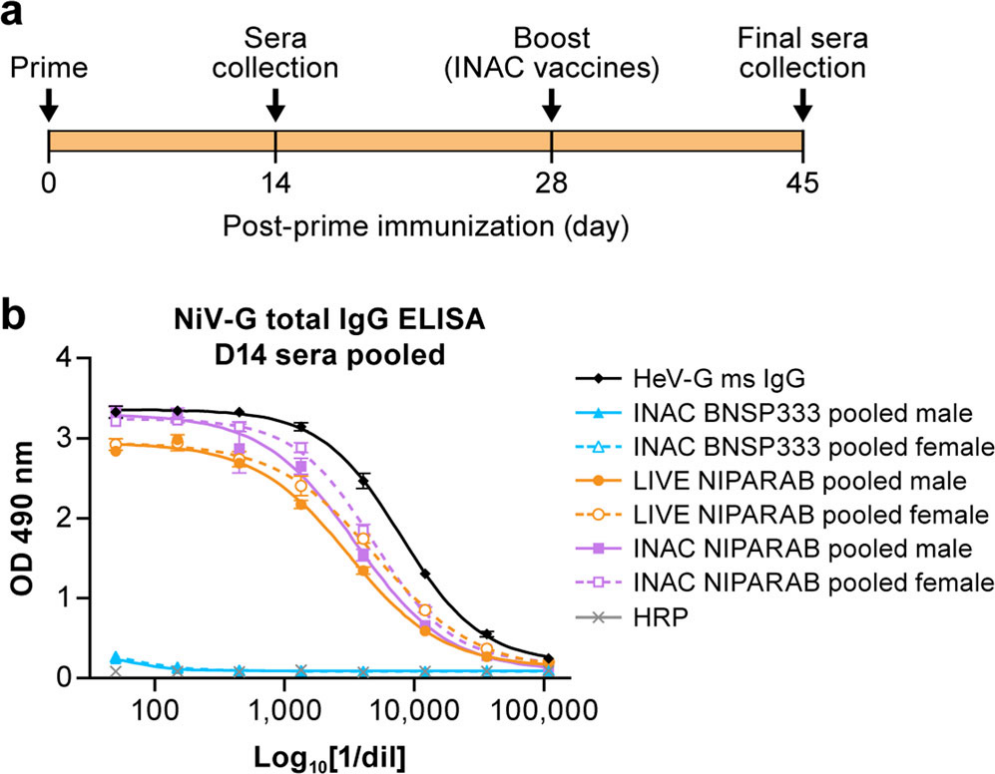
Primary humoral responses to NiV G in murine model of NIPARAB immunization. **a** Experimental timeline for immunization of C57BL/6 mice (*n* = 10, 5 males and 5 females per group). C56BL/6 mice were immunized with 1 dose of 10^5^ ffu live virus (Day 0) or 2 doses of 10 μg inactivated virus (Days 0 and 28). Sera were collected on days 14 and 45 and analyzed by ELISA to determine humoral response. **b** ELISA of the primary NiV G specific responses in pooled sera from either male or female mice in each vaccination group, 14 days after the initial immunization (prime). Error bars represent standard deviation from the mean of three replicate values

To analyze the humoral response to NiV G elicited by NIPARAB, groups of ten C57BL/6 mice (5 males, 5 females) were intramuscularly (i.m.) immunized with one dose (1E5 ffu) of live or 2 doses (10 ug each) of either INAC NIPARAB or INAC BNSP333 (Fig. 4a). Sera were collected at days 14, 28, and 45 and analyzed in an indirect ELISA using soluble NiV G as capture antigen to quantitatively determine NiV G-specific antibody titers. As a positive control in these assays, we included pooled antisera from a group of mice immunized i.n. with live recombinant VSV expressing codon-optimized HeV G, which was shown to be cross-reactive toward NiV G in both Western blot (Fig. 2b) and IF (Fig. 1b). Robust seroconversion was achieved as early as day 14 after a single vaccination in groups immunized with either live or INAC NIPARAB but not in the control group receiving INAC BNSP333 (Fig. 4b).

**Fig. 5:**
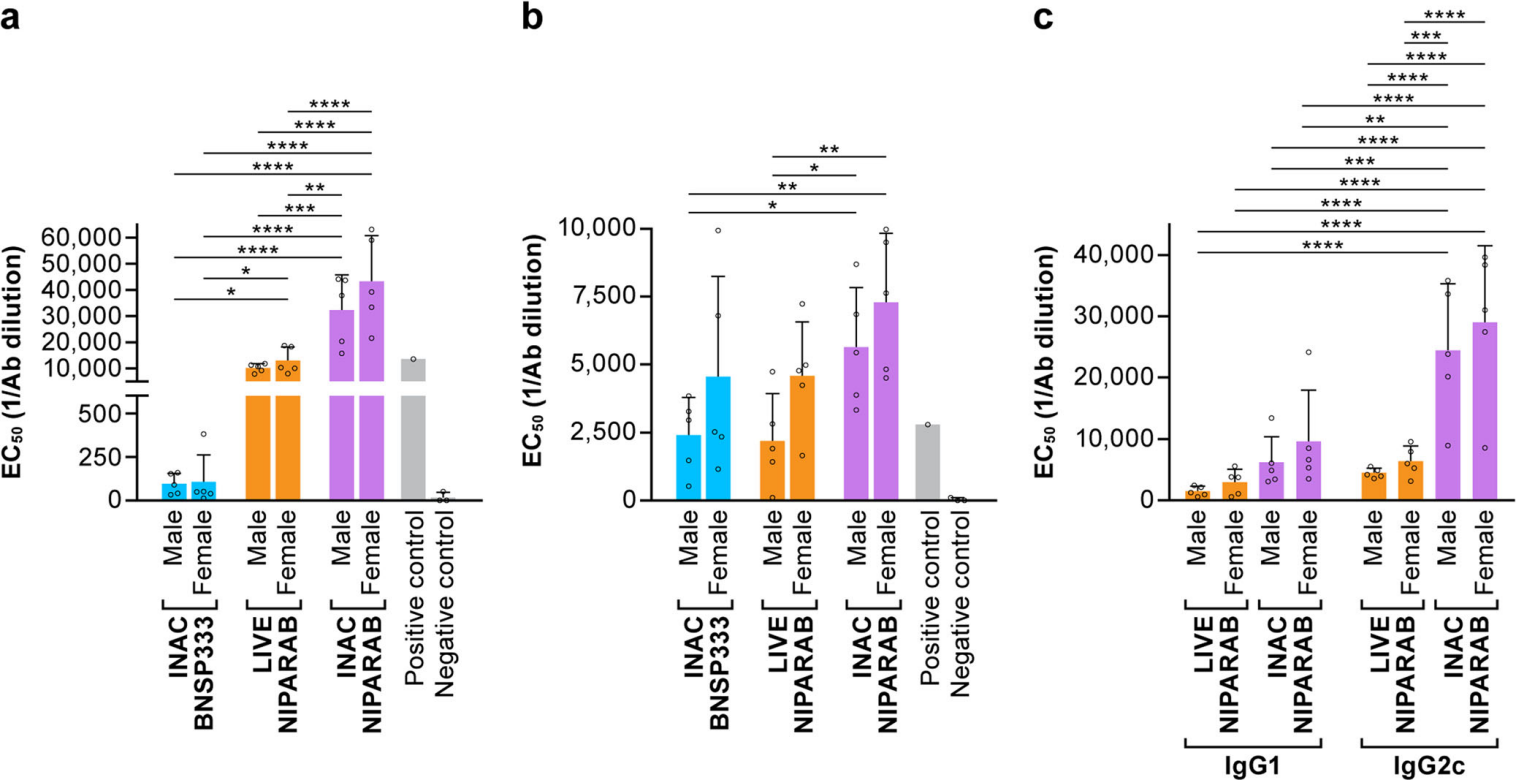
Analysis of terminal sera from NIPARAB immunized mice. **a**, **b** Average EC50 values (bars) were derived from ELISA curves measuring either total NiV G IgG (left panel) or RABV G (middle panel) from individual mice (open circles) from each vaccination group. Statistical significance was performed using 2-way ANOVA followed by uncorrected Fisher’s LSD to compare immunization groups (*p < 0.05, **p < 0.01, ***p < 0.001, ****p < 0.0001). **c** IgG1 and IgG2 isotype responses in final sera (day 45) from live or inactivated NIPARAB-immunized mice were assessed by ELISA at day 45 and average EC50 values (bars) were determined based on individual ELISA curves (open circles). Statistical significance was performed using 3-way ANOVA followed by uncorrected Fisher’s LSD test to compare immunization groups (*p < 0.05, **p < 0.01, ***p < 0.001, ****p < 0.0001). Error bars represent standard deviation from the mean of three replicate values

Approximately 2 weeks after completion of the prime-boost immunization schedule (day 45), we characterized final sera by ELISA to analyze the persistence of the NiV G-specific humoral response. Average EC50 values for each group were calculated based on 4-parameter logistic regression curves for each animal. All mice vaccinated with live or INAC NIPARAB elicited substantially, higher titers of anti-NiV G antibodies compared with BNSP333-vaccinated negative control animals, which displayed background levels of reactivity (i.e., EC50 values were undetermined) (Fig. 5a, Table 1). Furthermore, male and female mice immunized with INAC NIPARAB had significantly higher NiV G-specific antibody titers than either male or female mice receiving live NIPARAB (Fig. 5a, Table 1). All groups of mice seroconverted toward RABV G, and both male and female animals in the INAC NIPARAB group showed significantly higher titers of RABV G-specific antibodies that either male or female animals from both the negative control and live NIPARAB groups (Fig. 5b, Table 2).

**Table 1:** Anti-Nipah G IgG responses

Multiple comparisons: uncorrected Fisher’s LSD			Summary	Individual *P* value
Male:INAC NIPARAB	vs.	Male:LIVE NIPARAB	***	0.0009
Male:INAC NIPARAB	vs.	Male:INAC BNSP333	****	<0.0001
Male:INAC NIPARAB	vs.	Females:LIVE NIPARAB	**	0.0031
Male:INAC NIPARAB	vs.	Females:INAC BNSP333	****	<0.0001
Male:LIVE NIPARAB	vs.	Females:INAC NIPARAB	****	<0.0001
Male:INAC BNSP333	vs.	Females:INAC NIPARAB	****	<0.0001
Male:INAC BNSP333	vs.	Females:LIVE NIPARAB	*	0.036
Females:INAC NIPARAB	vs.	Females:LIVE NIPARAB	****	<0.0001
Females:INAC NIPARAB	vs.	Females:INAC BNSP333	****	<0.0001
Females:LIVE NIPARAB	vs.	Females:INAC BNSP333	*	0.0361

*p < 0.05, **p < 0.01, ***p < 0.001, ****p < 0.0001

**Table 2:** Anti-rabies G IgG responses

Multiple comparisons: uncorrected Fisher’s LSD			Summary	Individual *P* value
Male:INAC NIPARAB	vs.	Male:LIVE NIPARAB	*	0.031
Male:INAC NIPARAB	vs.	Male:INAC BNSP333	*	0.0423
Male:LIVE NIPARAB	vs.	Females:INAC NIPARAB	**	0.0024
Male:INAC BNSP333	vs.	Females:INAC NIPARAB	**	0.0035

*p < 0.05, **p < 0.01

From previous studies in our lab with RABV-vectored vaccines against hemorrhagic fever viruses, we discovered that antibody quality informs survival and that a Th1-biased humoral response is beneficial for controlling infection after lethal challenge.^37,38^ In C57BL/6 mice, skewing toward production of IgG2c antibodies is indicative of a Th1-type response whereas generation of IgG1 antibodies suggest a Th2-type immunity. We assessed relative titers of IgG2c and IgG1 antibodies in NiV G-specific sera from animals immunized with either live or INAC NIPARAB by isotype ELISA. Whereas there was no significant difference (*p* > 0.05) in titers of IgG2c vs. IgG1 antibodies in either male or female mice receiving live NIPARAB, both male and female mice receiving INAC NIPARAB had significantly higher titers of IgG2c vs. IgG1 (Fig. 5c, Table 3). Moreover, INAC NIPARAB mice had significantly higher titers of IgG2c (but not IgG1) antibodies compared to the live vaccine group, regardless of gender (Fig. 5c, Table 3).

**Table 3:** Anti-Nipah G IgG isotype responses

Multiple comparisons: uncorrected Fisher’s LSD			Summary	Individual *P* value
IgG1:Male INAC NIPARAB	vs.	IgG2c:Male INAC NIPARAB	***	0.0002
IgG1:Male INAC NIPARAB	vs.	IgG2c:Females INAC NIPARAB	****	<0.0001
IgG1:Females INAC NIPARAB	vs.	IgG2c:Male INAC NIPARAB	**	0.0016
IgG1:Females INAC NIPARAB	vs.	IgG2c:Females INAC NIPARAB	****	<0.0001
IgG1:Male LIVE NIPARAB	vs.	IgG2c:Male INAC NIPARAB	****	<0.0001
IgG1:Male LIVE NIPARAB	vs.	IgG2c:Females INAC NIPARAB	****	<0.0001
IgG1:Females LIVE NIPARAB	vs.	IgG2c:Male INAC NIPARAB	****	<0.0001
IgG1:Females LIVE NIPARAB	vs.	IgG2c:Females INAC NIPARAB	****	<0.0001
IgG2c:Male INAC NIPARAB	vs.	IgG2c:Male LIVE NIPARAB	****	<0.0001
IgG2c:Male INAC NIPARAB	vs.	IgG2c:Females LIVE NIPARAB	***	0.0002
IgG2c:Females INAC NIPARAB	vs.	IgG2c:Male LIVE NIPARAB	****	<0.0001
IgG2c:Females INAC NIPARAB	vs.	IgG2c:Females LIVE NIPARAB	****	<0.0001

**p < 0.01, ***p < 0.001, ****p < 0.0001

### Vaccine-induced NiV G neutralizing antibody titers

**Fig. 6:**
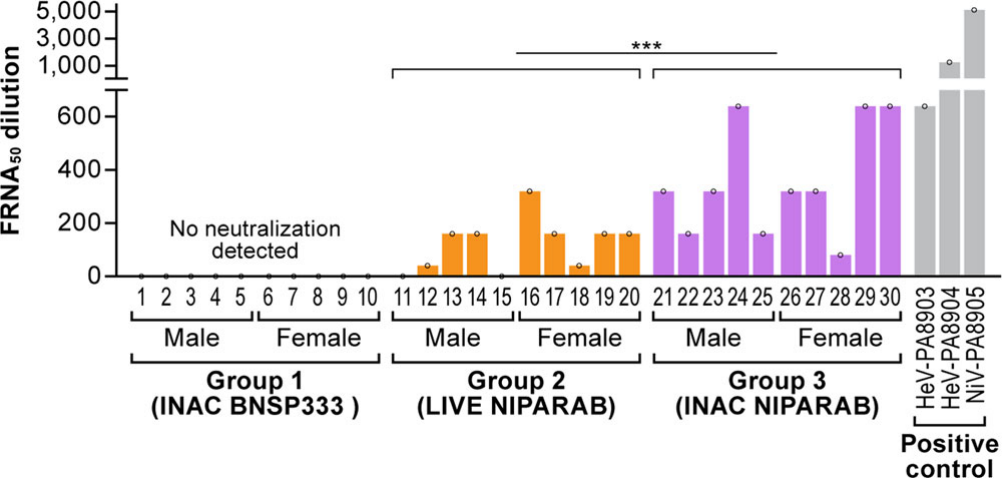
Vaccine-induced neutralization titers in vitro. Sera from mice in our immunization study (*n* = 10 per group) were analyzed in a Fluorescence Reduction Neutralization Assay 50 (FRNA50) with one FRNA50 value per animal. *Y*-axis gives the antibody dilution required to reduce infectivity by 50%. Both NiV G and HeV G rabbit polyclonal antisera were used as positive controls (gray bars). Statistical significance between groups was performed using ordinary one-way ANOVA followed by uncorrected Fisher’s LSD test to compare immunization groups. Non-parametric Kruskal-Wallis test followed by uncorrected Dunn’s multiple comparisons test was used to compare statistical significance based on gender (*p < 0.05, **p < 0.01, ***p < 0.001, ****p < 0.0001)

Neutralizing antibodies (nAbs) are a correlate of protection against many enveloped viruses including RABV, respiratory syncytial virus, and dengue fever virus.^39–41^ To evaluate the capacity of antibodies elicited by the NIPARAB vaccine to neutralize NiV, we employed an in vitro fluorescence reduction neutralization assay 50 (FRNA50).^15^ FRNA50 was performed as described in Materials and Methods. (Fig. 6, gray bars). HeV-specific sera effectively neutralized NiV, hence demonstrating that HeV (G-specific) antibodies not only bind to NiV G but also possess antiviral effector function. No neutralization was observed in sera from negative control mice immunized with INAC BNSP333, which is expected since these mice did not have any NiV G-specific antibody titers in ELISA (Fig. 6, Group 1). However, sera from all mice immunized with INAC NIPARAB developed nAbs, although titers were variable between individual animals (Fig. 6, Group 3). Similarly, mice vaccinated with live NIPARAB generated NiV G-specific nAbs, except for 2 male animals for which no nAb titers were detected (Fig. 6, Group 2). On account of these two animals, there were significant differences in nAb titers between live NIPARAB-immunized males and corresponding females (*p < 0.05), as well as when compared with both males and females immunized with INAC NIPARAB (*p < 0.05). Overall, animals in the INAC NIPARAB cohort had significantly higher titers of nAbs than animals in the corresponding live vaccine group (***p < 0.001). These results suggest that neutralization may be an important component of vaccine-induced protection by NIPARAB and that 2 doses of the killed vaccine are more immunogenic than inoculation with live vaccine regarding generation of NiV-specific nAbs. However, a challenge study in animals vaccinated with NIPARAB needs to be conducted to determine if nAbs correlate with survival.

## Discussion

Nipah virus is one of eight viruses designated by the World Health Organization (WHO) as a priority pathogen due to its potential to cause an international epidemic in the absence of effective drugs or vaccines.^42^ Although outbreaks have been sporadic, mortality rates in humans have been high (40 to 75%).^1,3^ Furthermore, there is a significant economic burden to pig farmers in endemic regions whose livestock must be culled in response to an outbreak.^43^ We have described the development of a killed recombinant rabies-vectored NiV vaccine, NIPARAB, for eventual use in humans to prevent NiV disease. We have also characterized a live attenuated NIPARAB vaccine to be considered for use in wildlife to curb transmission from the natural reservoir (pteropid bats) to secondary reservoirs (e.g., pigs) and diminish spillover events to humans.

Based on studies in African green monkeys (AGMs) comparing the pathogenic differences between the Malaysian (NiV_M_) and Bangladesh (NiV_B_) strains of NiV under identical experimental conditions, we decided to choose NiV_B_ as the immunogen in our candidate NIPARAB vaccine. NiV_B_ caused 100% mortality in infected AGMs while NiV_M_ caused only 50% mortality. Furthermore, histopathology in NiV_B_-infected AGMs showed more severe lung and spleen phenotypes and a shorter therapeutic window for human monoclonal antibody treatment than NiV_M_-infected counterparts.^44^ Since there is a 95% amino acid sequence homology between the glycoproteins of these two strains,^19^ an effective vaccine against the more highly pathogenic NiV_B_ strain would likely protect against exposure to NiV_M_. In fact, this has been demonstrated experimentally: ferrets vaccinated with a live attenuated vesicular stomatitis virus (VSV) vaccine expressing NiV G from the Bangladesh strain were 100% protected against heterologous challenge with a Malaysian isolate of NiV.^45^ Studies have also successfully utilized the F protein of NiV to develop protective vaccines against both strains and HeV,^15,19^ but we decided to target the glycoprotein to block virion-receptor binding, an entry step that occurs before membrane fusion.

Our results indicated that both live and inactivated NIPARAB elicited strong humoral immunity in mice, characterized by high titers of antibodies against NiV G (Fig. 5a) and induction of potently neutralizing antibodies against wildtype Malaysian NiV strain (Fig. 6). Antibodies generated by INAC NIPARAB were mainly of IgG2c isotype, suggesting a Th1-biased response. In antiviral defense, Th1-type antibodies are known to participate in non-neutralizing effector functions, such as NK cell-mediated antibody dependent cytotoxicity (ADCC) or macrophage/monocyte-mediated antibody dependent cellular phagocytosis (ADCP).^46–49^ It is possible that these other mechanisms contribute to vaccine-induced protection with INAC NIPARAB in addition to classic neutralization.

INAC NIPARAB offers several advantages over other NiV vaccines currently under development. Since our candidate vaccine for humans is chemically killed, viral replication is completely abrogated, rendering our vaccine potentially safer than NiV vaccines that use a live vector (e.g., live-attenuated rVSV-ZEBOV-GP-NiV_M_G), especially in susceptible populations like immunocompromised individuals, pregnant women, or children. A live virus vaccine might be better intended for use in wildlife. Indeed, the rabies strain upon which our recombinant NIPARAB is based (SAD B19) has been successfully used as a live oral vaccine for wildlife in Europe.^50^ Our data have shown that live NIPARAB does not cause pathogenicity in mice, even when administered at a high dose (5.75log_10_ ffu) intranasally, and has the added benefit of bivalency toward rabies, a devastating pathogen that widely affects both humans and wildlife in the same geographic regions where NiV outbreaks occur. Moreover, INAC NIPARAB is immunogenic after a single dose of unadjuvanted vaccine (Fig. 4b), and titers remain elevated even one month after secondary unadjuvanted immunization. By contrast, soluble NiV and HeV glycoprotein subunit and VLP-based vaccines require one or more doses of adjuvant to be immunogenic.^15,19^ Furthermore, INAC NIPARAB does not have to contend with the issue of pre-existing vector immunity in human cohorts like measles-vectored NiV vaccines.^12^ Previous studies have shown additive immunity with subsequent vaccinations of rabies-vectored inactivated vaccines.^7,37,38,51^

The studies described here demonstrate the potential for a RABV platform for the development of a safe and effective NiV vaccine. Future studies will test NIPARAB protection in an animal model susceptible to NiV, such as Syrian golden hamsters, and will also establish the minimum protective dose and capacity to reduce NiV disease severity and/or mortality. Furthermore, protection against HeV challenge will be assessed since there is wide evidence for sera cross reactivity between these two henipaviruses.^52–54^

## Methods

### cDNA construction of vaccine vectors

We inserted codon-optimized Nipah virus glycoprotein gene G (Bangladesh strain, GenBank: AY988601.1) between the N and P genes of the parental BNSP333 rabies vector using BsiWI and NheI restriction sites^(16)^. Codon bias optimization for human codon use was carried out by GenScript Inc. The resulting plasmid was designated BNSP333-coNiV-G (NIPARAB), and the correct sequence of the plasmid was confirmed by sequencing using primers targeting the region between the N and P genes.

### Recovery of recombinant vectors

X-tremeGENE 9 transfection reagent (Roche Diagnostics) in Opti-MEM was used to transfect full-length viral cDNA clones along with support plasmids bearing RABV N, P, G, and L genes under the control of a T7 promoter and a plasmid expressing T7 RNA polymerase into BSR cells on 6-well plates as described previously.^31^ Successful recovery was determined by a rabies focus-forming assay. Briefly, seven days after transfection, supernatant from each transfected well of the 6-well plate was transferred to duplicate wells of a 12-well plate seeded with VERO cells. Forty-eight hours later, cells in the 12-well plate were fixed with 80% acetone and stained with a FITC-conjugated antibody against RABV N (Fujirebio Diagnostics, Inc). Fluorescence microscopy was used to observe the appearance of viral foci, indicative of recovered, infectious recombinant RABV.

### Sucrose purification and inactivation of virus particles

NIPARAB was grown large-scale by infecting VERO cells in a 2-stack plate at MOI = 0.001. The supernatant was collected every 4 days for 6 harvests. Harvests were titered using rabies focus-forming assay^55^ and harvest 4–6 were pooled and concentrated 9× in a stirred 300-ml ultrafiltration cell (Millipore). Concentrated supernatant was then centrifuged for 2 h at 76,755×*g* through a 20% sucrose cushion using SW32 Ti rotor (Beckman, Inc.) to pellet virus particles. Virion pellets were resuspended in phosphate-buffered saline (PBS), and protein concentrations were determined using a bicinchoninic acid (BCA) assay kit (Pierce). The virus particles were chemically inactivated with β-propiolactone (BPL) at a dilution of 1:2000 overnight at 4 °C. BPL in the virus preparation was inactivated the next day by hydrolysis at 37 °C for 30 min. The absence of infectious particles was verified by inoculating VERO cells in a T25 vessel with 10 μg of BPL-inactivated virus for two passages. Inoculated cells were fixed and stained with FITC-conjugated anti-RABV N mAb and visualized by fluorescence microscopy for the presence of foci of infection.

### Immunofluorescence testing of the vaccine

VERO cells were plated onto 12 well plates with 3E5 cells with 15 mm circular diameter coverslips inserted and then incubated overnight at 37 °C. The next day the wells were infected at an MOI of 0.01 in 500 μL of serum-free media (OptiPro) per well with NIPARAB or BNSP333-cover-G mixed by rocking and then stored at 34 °C for 48 h. After 48 h, cells were washed with 1 mL of 1× PBS, then fixed with 500 μL of 2% paraformaldehyde (PFA) diluted in PBS for 15 min at room temperature. PFA was removed by aspiration and cells washed 3× with 1× PBS. 1 mL of blocking solution (4% fetal bovine serum [FBS] in PBS) was added to each well for 1 h at room temperature while on the shaker. Blocking solution was aspirated off, then 500 μL of dual primary stain (1:250 dilution of anti-RABV G human mAb 4C12 at 4 mg/mL (Dr. Scott Dessain, Lankenau Institute for Medical Research, Wynnewood, PA) plus 1:200 dilution of mouse sera) in 2% FBS was added for 1 h while rocking. Cells were washed four times with 1× PBS and then incubated with 500 μL of a 1:250 dilution of both anti-mouse Cy3 (Jackson ImmunoResearch) and anti-human Cy2 (Jackson ImmunoResearch) secondary antibodies containing Cy2 and Cy3 dyes and incubated at room temperature for 45 min. Cells were washed 5 times with 1× PBS and then cells were mounted onto slides with the mounting solution containing DAPI (Abcam) with the coverslips face down onto the slide and stored overnight at room temperature for visualization by confocal microscopy the following day.

### Virus characterization

Sucrose-purified virus particles were denatured with urea buffer (125 mM Tris-HCl [pH 6.8], 8 M urea, 4% sodium dodecyl sulfate, 5% β-mercaptoethanol, 0.02% bromophenol blue, Thermo Fisher Scientific) at 95 °C for 5 min. *Stained protein gel*: 8 μg of particles were resolved by 10% SDS–PAGE and thereafter stained overnight with SYPRO Ruby for total protein analysis. *Western blot*: 4 μg of particles were resolved on a 10% SDS-PAGE gel and transferred onto a nitrocellulose membrane in Towbin buffer (192 mM glycine, 25 mm Tris, 20% methanol, Thermo Fisher Scientific) for Western blot analysis. The nitrocellulose membrane was then blocked in TBST (100 mM Tris-HCl [pH 7.9], 150 mM NaCl, 0.05% Tween 20, Thermo Fisher Scientific) containing 5% dried milk (MilliporeSigma) at room temperature for 1 h. After blocking, the membrane was incubated overnight with rabbit polyclonal HeV G antisera (Dr. Christopher Broder, Uniformed Services University, Bethesda, MD) at a dilution of 1:1000 in antibody diluent (1% bovine serum albumin (BSA) in PBS with 0.1% Tween-20). After washing, the blot was incubated for 1 h with donkey anti-rabbit IgG conjugated to horseradish peroxidase (HRP) at a 1:20,000 in antibody diluent. Bands were developed with SuperSignal West Dura Chemiluminescent substrate (Pierce). The SDS-PAGE gel and corresponding Western blot in Fig. 4 derived from the same experiment and were processed in parallel.

### Pathogenicity and immunogenicity studies

#### (i) Animal ethics statement

This study was carried out in strict adherence to recommendations described in the *Guide for the Care and Use of Laboratory Animals*,^56^ as well as guidelines of the National Institutes of Health, the Office of Animal Welfare, and the United States Department of Agriculture. All animal work was approved by the Institutional Animal Care and Use Committee (IACUC) at Thomas Jefferson University (animal protocols 00990 and 01526). All procedures were carried out under isoflurane anesthesia by trained personnel, under the supervision of veterinary staff. Mice were housed in cages, in groups of 5, under controlled conditions of humidity, temperature, and light (12-h light/12-h dark cycles). Food and water were available ad libitum.

#### (ii) Immunizations

Three groups of 6-week-old to 8-week-old C56BL/6 mice were immunized intramuscularly with 10 μg of virus particles in a total volume of 100 μL (50 μL per hindlimb). The three groups were as follows: inactivated NIPARAB, live NIPARAB, and inactivated BNSP333. Each group consisted of 5 male and 5 female mice. Mice receiving inactivated vaccine were given two doses, once on day 0 and once on day 28, while mice receiving the live NIPARAB vaccine were only immunized once, on day 0.

#### (iii) Pathogenicity experiments

Three groups of 6-week-old to 8-week-old C56BL/6 mice were intranasally infected with 10 μL of 5.6 × 10^5^ ffu of live virus (SPBN, BNSP333, or NIPARAB). Each group consisted of 5 male and 5 female mice. The mice were monitored for signs of disease such as ruffled fur, ataxia, and disorientation and weighed until day 40. Mice that lost more than 20% of their original weight were considered to have reached the endpoint and were euthanized.

### Production of HA-tagged NiV G

Subconfluent T175 flasks of 293 T cells (human kidney cell line) were transfected with a eukaryotic expression vector (pDisplay) encoding amino acids 71 to 602 of the head and stalk domains of codon-optimized NiV G (Bangladesh strain) fused to an N-terminal hemagglutinin (HA) peptide. Supernatant was collected 48 h after transfection, clarified by centrifugation, and filtered through a 0.45 μm filter before being loaded onto an equilibrated anti-HA agarose column (Pierce) containing a 2.5 ml agarose bed volume. The supernatant was allowed to bind to the column overnight at 4 °C. The next day, the column was washed with 10-bed volumes of TBST (TBS with 0.05% Tween 20) and 2-bed volumes of TBS, and bound HA-coNiV-G was eluted with 5 ml of 250 μg/ml HA peptide in TBS. Fractions were collected and analyzed for the presence of HA-coNiV-G by western blotting with monoclonal anti-HA antibody (Sigma) prepared in 5% BSA-TBST. Peak fractions were pooled and dialyzed against PBS in 10,000 molecular weight cutoff dialysis cassettes (MWCO) (Thermo Scientific) to remove excess HA peptide. After dialysis, the protein was quantified by BCA and frozen in aliquots at −80 °C.

### RABV and NiV G responses by ELISA

Sera from immunized mice were collected by retro-orbital eye bleed under isoflurane anesthesia on days 0, 14, and 45, and samples were tested for immunogenicity by indirect ELISA using N-terminus HA-tagged soluble recombinant protein for antibody capture. We tested individual mouse sera, as well as pooled sera for the presence of total IgG specific to NiV G and RABV G. To test for anti-NiV G humoral responses, we produced soluble NiV G (sNiV-G) as described above. sNiV-G was diluted in coating buffer (50 mM Na_2_CO_3_ [pH 9.6]) at a concentration of 500 ng/mL and then plated in 96-well ELISA MaxiSorp plates (Nunc) at 100 μL in each well. RABV-G was also resuspended in coating buffer at a concentration of 500 ng/mL and then plated in 96-well ELISA MaxiSorp plates (Nunc) at 100 μl per well. After overnight incubation at 4 °C, plates were washed three times with PBST (0.05% Tween 20 in 1× PBS) and incubated for 1 h at room temperature with blocking buffer (5% dry milk powder in 1× PBST) in a volume of 250 μl per well. The plates were then washed three times with PBST and incubated overnight at 4 °C with 3-fold or 4-fold serial dilutions of sera from immunized mice in PBS containing 0.5% BSA. Plates were washed 3 times the next day, followed by the addition of horseradish peroxidase-conjugated goat anti-mouse-IgG (H + L) secondary antibody (1:10,000) (Jackson ImmunoResearch). After incubation for 2 h at room temperature, plates were washed 3 times with PBST, and 200 μl of *o*-phenylenediamine dihydrochloride (OPD) substrate (Sigma) was added to each well. The reaction was stopped by the addition of 50 μl of 3 M H_2_SO_4_ per well after 15 min. Optical density was determined at 490 nm (OD_490_).

### Fluorescence reduction neutralization assay (FRNA50)

FRNA50 was used to determine the highest serum dilution, which would reduce the infectivity of the virus by 50%. This assay (with minor modifications) was described in Walpita et al., 2017 (ref.^14^). Briefly, VERO E6 cells were seeded at 40,000 cells/well at 1 day prior to the experiment. Serum samples were heat inactivated for 60 min at 56 °C and serially diluted in serum-free DMEM. After incubation, diluted samples were mixed with 4000 PFU of NiV and incubated for 1 h at 37 °C. Serum-virus mixtures were added to cells’ monolayers and incubated at 37 °C/5% CO_2_ for 48 h before fixing with 10% NBF for 24 h. Immunofluorescence assay (IFA) was performed by permeabilizing of cells with 0.25% Triton X-100 in PBS for 5 min, blocking with 3% BSA for 30 min, staining with a NiV G protein-specific rabbit polyclonal antiserum (produced by ThermoFisher Scientific from NiV GP “293 FreeStyle Tet-NiV-sG”, a gift from Dr. Christopher C. Broder, Uniformed Services University) in 3% BSA diluted at 1:2000 at 37 °C for 60 min. In the final step of IFA, goat anti-rabbit IgG (H + L) secondary antibody, Alexa Fluor® 594 conjugate (Life Technologies), and Hoechst 33342 nucleic acid stain (ThermoFisher Scientific) were applied to the cells for 30 min at room temperature (1:2500 dilution each). Cells were washed with 1× PBS between each step, except for “blocking-primary antibody step”. The percentage of cells infected with NiV was detected using High Content Imaging System Operetta (PerkinElmer).

### Reporting Summary

Further information on experimental design is available in the Nature Research Reporting Summary linked to this article.

## Supplementary information

Reporting Summary

## Data Availability

All relevant data are available from the corresponding author upon reasonable request. Codon-optimized sequences of NiV G (Bangladesh) are derived from GenBank accession code: AY988601.1 and are available upon request.

## Supplementary information

**Supplementary Information** accompanies the paper on the *npj Vaccines* website (10.1038/s41541-019-0109-5).

## References

[1] Chua KB (2000). Nipah virus: a recently emergent deadly paramyxovirus. Science.

[2] Eaton BT, Broder CC, Middleton D, Wang LF (2006). Hendra and Nipah viruses: different and dangerous. Nat. Rev. Microbiol..

[3] Ang BSP, Lim TCC, Wang L (2018). Nipah Virus Infection. J. Clin. Microbiol..

[4] WHO. *Morbidity and Mortality Due to Nipah or Nipah-like Virus Encephalitis in WHO South-East Asia Region, 2001–2018.* http://www.searo.who.int/entity/emerging_diseases/links/morbidity-and-mortality-nipah-sear-2001-2018.pdf?ua=1 (WHO, 2018).

[5] Steffen DL, Xu K, Nikolov DB, Broder CC (2012). Henipavirus mediated membrane fusion, virus entry and targeted therapeutics. Viruses.

[6] Xu K, Broder CC, Nikolov DB (2012). Ephrin-B2 and ephrin-B3 as functional henipavirus receptors. Semin. Cell Dev. Biol..

[7] Kurup D, Wirblich C, Feldmann H, Marzi A, Schnell MJ (2015). Rhabdovirus-based vaccine platforms against henipaviruses. J. Virol..

[8] Negrete OA, Chu D, Aguilar HC, Lee B (2007). Single amino acid changes in the Nipah and Hendra virus attachment glycoproteins distinguish ephrinB2 from ephrinB3 usage. J. Virol..

[9] Lam SK (2003). Nipah virus--a potential agent of bioterrorism?. Antiviral. Res..

[10] Yoneda M (2013). Recombinant measles virus vaccine expressing the Nipah virus glycoprotein protects against lethal Nipah virus challenge. PLoS ONE.

[11] Hilleman MR (2001). Current overview of the pathogenesis and prophylaxis of measles with focus on practical implications. Vaccine.

[12] Knuchel MC (2013). Relevance of a pre-existing measles immunity prior immunization with a recombinant measles virus vector. Hum. Vaccin. Immunother..

[13] Reisinger EC (2019). Immunogenicity, safety, and tolerability of the measles-vectored chikungunya virus vaccine MV-CHIK: a double-blind, randomised, placebo-controlled and active-controlled phase 2 trial. Lancet.

[14] Grgacic EV, Anderson DA (2006). Virus-like particles: passport to immune recognition. Methods.

[15] Walpita P (2017). A VLP-based vaccine provides complete protection against Nipah virus challenge following multiple-dose or single-dose vaccination schedules in a hamster model. NPJ Vaccines.

[16] DeBuysscher BL, Scott D, Marzi A, Prescott J, Feldmann H (2014). Single-dose live-attenuated Nipah virus vaccines confer complete protection by eliciting antibodies directed against surface glycoproteins. Vaccine.

[17] Prescott J (2015). Single-dose live-attenuated vesicular stomatitis virus-based vaccine protects African green monkeys from Nipah virus disease. Vaccine.

[18] Lo MK (2014). Single-dose replication-defective VSV-based Nipah virus vaccines provide protection from lethal challenge in Syrian hamsters. Antiviral. Res..

[19] Satterfield BA, Dawes BE, Milligan GN (2016). Status of vaccine research and development of vaccines for Nipah virus. Vaccine.

[20] Juan-Giner A., et al. Safety of the rVSV ZEBOV vaccine against Ebola Zaire among frontline workers in Guinea. *Vaccine* pii:S0264-410X(18)31246-5 (2018). 10.1016/j.vaccine.2018.09.009 30266489

[21] Metzger WG, Vivas-Martinez S (2018). Questionable efficacy of the rVSV-ZEBOV Ebola vaccine. Lancet.

[22] Levy Y (2018). Prevention of Ebola virus disease through vaccination: where we are in 2018. Lancet.

[23] Blaney JE (2011). Inactivated or live-attenuated bivalent vaccines that confer protection against rabies and Ebola viruses. J. Virol..

[24] Gomme EA, Wanjalla CN, Wirblich C, Schnell MJ (2011). Rabies virus as a research tool and viral vaccine vector. Adv. Virus Res..

[25] Schnell MJ (2000). Recombinant rabies virus as potential live-viral vaccines for HIV-1. Proc. Natl Acad. Sci. USA.

[26] Vos A (1999). An update on safety studies of SAD B19 rabies virus vaccine in target and non-target species. Epidemiol. Infect..

[27] Cenna J (2009). Replication-deficient rabies virus-based vaccines are safe and immunogenic in mice and nonhuman primates. J. Infect. Dis..

[28] Mebatsion T, Schnell MJ, Cox JH, Finke S, Conzelmann KK (1996). Highly stable expression of a foreign gene from rabies virus vectors. Proc. Natl Acad. Sci. USA.

[29] Coulon P, Ternaux JP, Flamand A, Tuffereau C (1998). An avirulent mutant of rabies virus is unable to infect motoneurons in vivo and in vitro. J. Virol..

[30] McGettigan JP (2003). Second-generation rabies virus-based vaccine vectors expressing human immunodeficiency virus type 1 gag have greatly reduced pathogenicity but are highly immunogenic. J. Virol..

[31] Schnell MJ, Mebatsion T, Conzelmann KK (1994). Infectious rabies viruses from cloned cDNA. EMBO J..

[32] Bonnafous P (2014). Treatment of influenza virus with beta-propiolactone alters viral membrane fusion. Biochim. Biophys. Acta.

[33] Maar D (2012). Cysteines in the stalk of the nipah virus G glycoprotein are located in a distinct subdomain critical for fusion activation. J. Virol..

[34] Liu Q (2015). Nipah virus attachment glycoprotein stalk C-terminal region links receptor binding to fusion triggering. J. Virol..

[35] Fischer K (2018). Indirect ELISA based on Hendra and Nipah virus proteins for the detection of henipavirus specific antibodies in pigs. PLoS ONE.

[36] Bossart KN (2002). Membrane fusion tropism and heterotypic functional activities of the Nipah virus and Hendra virus envelope glycoproteins. J. Virol..

[37] Abreu-Mota T (2018). Non-neutralizing antibodies elicited by recombinant Lassa–Rabies vaccine are critical for protection against Lassa fever. Nat. Commun..

[38] Blaney JE (2013). Antibody quality and protection from lethal Ebola virus challenge in nonhuman primates immunized with rabies virus based bivalent vaccine. PLoS Pathog..

[39] McGettigan JP (2010). Experimental rabies vaccines for humans. Expert. Rev. Vaccines.

[40] Katzelnick LC, Montoya M, Gresh L, Balmaseda A, Harris E (2016). Neutralizing antibody titers against dengue virus correlate with protection from symptomatic infection in a longitudinal cohort. Proc. Natl Acad. Sci. USA.

[41] Piedra PA, Hause AM, Aideyan L (2016). Respiratory Syncytial Virus (RSV): neutralizing antibody, a correlate of immune protection. Methods Mol. Biol..

[42] WHO. *List of Blueprint Priority Diseases*. https://www.who.int/blueprint/priority-diseases/en/ (WHO, 2018).

[43] Chua KB (1999). Fatal encephalitis due to Nipah virus among pig-farmers in Malaysia. Lancet.

[44] Mire CE (2016). Pathogenic differences between nipah virus bangladesh and malaysia strains in primates: implications for antibody therapy. Sci. Rep..

[45] Mire CE (2013). Single injection recombinant vesicular stomatitis virus vaccines protect ferrets against lethal Nipah virus disease. Virol. J..

[46] Forthal DN, Moog C (2009). Fc receptor-mediated antiviral antibodies. Curr. Opin. HIV AIDS.

[47] Leeansyah E, Wines BD, Crowe SM, Jaworowski A (2007). The mechanism underlying defective Fcgamma receptor-mediated phagocytosis by HIV-1-infected human monocyte-derived macrophages. J. Immunol..

[48] Pelegrin M, Naranjo-Gomez M, Piechaczyk M (2015). Antiviral monoclonal antibodies: can they be more than simple neutralizing agents?. Trends Microbiol..

[49] Liu Q (2017). Antibody-dependent-cellular-cytotoxicity-inducing antibodies significantly affect the post-exposure treatment of Ebola virus infection. Sci. Rep..

[50] Cliquet F (2015). In-depth characterization of live vaccines used in europe for oral rabies vaccination of wildlife. PLoS ONE.

[51] Wirblich C (2017). One-health: a safe, efficient, dual-use vaccine for humans and animals against middle east respiratory syndrome coronavirus and rabies virus. J. Virol..

[52] Zhu Z (2008). Exceptionally potent cross-reactive neutralization of Nipah and Hendra viruses by a human monoclonal antibody. J. Infect. Dis..

[53] Broder CC (2013). Passive immunization and active vaccination against Hendra and Nipah viruses. Dev Biol.

[54] Guillaume V (2009). Acute Hendra virus infection: analysis of the pathogenesis and passive antibody protection in the hamster model. Virology.

[55] Pulmanausahakul R, Li J, Schnell MJ, Dietzschold B (2008). The glycoprotein and the matrix protein of rabies virus affect pathogenicity by regulating viral replication and facilitating cell-to-cell spread. J. Virol..

[56] NIH. *Guide for the Care and Use of Laboratory Animals*. https://grants.nih.gov/grants/olaw/guide-for-the-care-and-use-of-laboratory-animals.pdf (Nationa Academies Press, 2011). 21595115

