# Publisher Correction: Rabies-based vaccine induces potent immune responses against Nipah virus

**DOI:** 10.1038/s41541-019-0112-x

**Published:** 2019-05-13

**Authors:** Rohan Keshwara, Thomas Shiels, Elena Postnikova, Drishya Kurup, Christoph Wirblich, Reed F. Johnson, Matthias J. Schnell

**Affiliations:** 1 Department of Microbiology and Immunology, Sidney Kimmel Medical College at Thomas Jefferson University, Philadelphia, PA 19107 USA; 2 Integrated Research Facility, National Institute of Allergy and Infectious Diseases, National Institutes of Health, Fort Detrick, MD 21702 USA; 3 Emerging Viral Pathogens Section, National Institute of Allergy and Infectious Diseases, National Institutes of Health, Bethesda, MD 20892 USA; 4 Jefferson Vaccine Center, Sidney Kimmel Medical College at Thomas Jefferson University, Philadelphia, PA 19107 USA

**Correction to:** *npj Vaccines* 10.1038/s41541-019-0109-5, Published online 15 April 2019

In the original published version of this Article, the figure legends were attached to the wrong figures. The legend for Fig. 2 was moved to Fig. 4, the legend for Fig. 3 was moved to Fig. 2, and the legend for Fig. 4 was moved to Fig. 3. The PDF and HTML versions of the Article have been corrected.

